# A Simplified Machine Learning Model for Predicting Reduced Kidney Function in Thai Patients with Type 2 Diabetes: A Retrospective Study

**DOI:** 10.3390/jcm14134735

**Published:** 2025-07-04

**Authors:** Wanjak Pongsittisak, Swangjit Suraamornkul

**Affiliations:** 1 Nephrology and Renal Replacement Therapy Division, Department of Internal Medicine, Faculty of Medicine Vajira Hospital, Navamindradhiraj University, Bangkok 10300, Thailand; 2 Vajira Big Data and Machine Learning Applied for Healthcare (VBaM4H) Research Group, Bangkok 10300, Thailand; 3 Faculty of Medicine, Vajira Hospital, Navamindradhiraj University, Bangkok 10300, Thailand; swangjit@nmu.ac.th

**Keywords:** type 2 diabetes, chronic kidney disease, risk prediction, machine learning, Thailand

## Abstract

**Background:** Chronic kidney disease (CKD) is a prevalent complication among individuals with type 2 diabetes (T2D), posing significant diagnostic challenges in resource-limited settings due to infrequent testing and missed hospital visits. This study aimed to develop a simple, effective ML model to identify T2D patients at high risk for reduced kidney function. **Methods:** We retrospectively analyzed data from 3471 T2D patients collected over a ten-year period at a university hospital in Bangkok, Thailand. Two models were developed using readily available clinical features: one including hemoglobin A1c (HbA1c) levels (the “with-HbA1c” model) and one excluding HbA1c levels (the “non–HbA1c” model). Three tree-based ML algorithms—decision tree, random forest, and extreme gradient boosting (XGBoost) algorithms—were employed. The outcome label was CKD, defined as an estimated Glomerular Filtration Rate (eGFR) < 60 mL/min/1.73 m^2^ that persisted for more than 90 days. The model performance was evaluated using the AUROC. The feature importance was assessed using Shapley additive explanations (SHAP). **Results:** The XGBoost algorithm demonstrated a strong predictive performance. The “with-HbA1c” model achieved an AUROC of 0.824, while the “non–HbA1c” model attained a comparable AUROC of 0.819. Both models were well-calibrated. SHAP analysis identified age, HbA1c, and systolic blood pressure as the most influential predictors. **Conclusions:** Our simplified, interpretable ML models can effectively stratify the risk of reduced kidney function in patients with T2D using minimal, routine data. These models represent a promising step toward integration into clinical practice, such as through EHR-based alerts or patient-facing mobile applications, to improve early CKD detection, particularly in resource-limited settings.

## 1. Background

Type 2 diabetes (T2D) is a global health challenge driven by its profound microvascular and macrovascular complications. The cornerstone of this pathology is chronic hyperglycemia, which induces endothelial dysfunction, oxidative stress, and inflammation, leading to widespread vascular damage, from cardiovascular disease (CVD) to peripheral vascular ulcers [1,2]. This process results in a constellation of severe clinical outcomes, including chronic kidney disease (CKD) and CVD, which frequently coexist and synergistically worsen patient prognoses [3]. Globally, T2D is the leading cause of CKD, with estimates suggesting that approximately 40% of individuals with T2D will develop kidney disease during their lifetimes [4,5]. The progression of diabetic kidney disease not only leads to End-Stage Kidney Disease (ESKD)—a devastating condition requiring costly kidney replacement therapies—but also acts as a potent risk multiplier for cardiovascular events, which remain the primary cause of mortality in this population [6,7]. In Thailand, the prevalence of T2D has surged to nearly 10% of the adult population, placing a substantial and growing burden on the national healthcare system [8]. National data confirm that a significant proportion of Thai patients with T2D, approximately 40%, exhibit signs of reduced kidney function [9]. The economic toll is multifaceted, comprising high direct costs of care, lost productivity, and a severely diminished quality of life [10,11].

In response to this rising burden, healthcare is increasingly leveraging new technologies to improve patient management [12]. Mobile health (mHealth) interventions, for instance, have demonstrated success in empowering self-care for patients with T2D, heart failure, and CKD [13,14,15,16]. Within this paradigm, machine learning (ML) has emerged as a powerful tool for risk stratification and clinical decision support, capable of identifying high-risk individuals long before overt complications arise [17,18]. Despite this promise, the clinical adoption of many ML models for CKD prediction is hampered by limitations such as their low interpretability and reliance on specialized variables not available in primary care, making them impractical for widespread screening [19]. Furthermore, models developed on Western populations may not be well-calibrated for Thai patients due to genetic and lifestyle differences. A critical barrier is the inconsistent availability of glycated hemoglobin (HbA1c), a key predictor whose absence should not prevent initial risk assessment. A tool that functions reliably without it would possess immense clinical utility.

Therefore, this study was designed to address these gaps by developing simplified, transparent, and practical prediction models using a retrospective cohort of Thai patients with T2D. The primary objective was to develop and validate an ML model incorporating HbA1c and routine clinical variables to predict the risk of CKD. For the purposes of this study, CKD was defined operationally using only the eGFR criterion: an eGFR < 60 mL/min/1.73 m^2^ persisting for more than 90 days. The secondary objectives were to develop and validate a more accessible model that excludes HbA1c to ensure broad utility and accessibility across diverse clinical settings.

## 2. Methods

### 2.1. Data Source

This was a retrospective study using data collected from the adult diabetes center at a university hospital in Bangkok, Thailand, for the period between 1 January 2005 and 31 December 2014. Personal identifiers were removed; patient identification was through a unique registry number. The registry included patients who were receiving follow-up care for T2D at our center.

### 2.2. Study Populations

**Figure 1:**
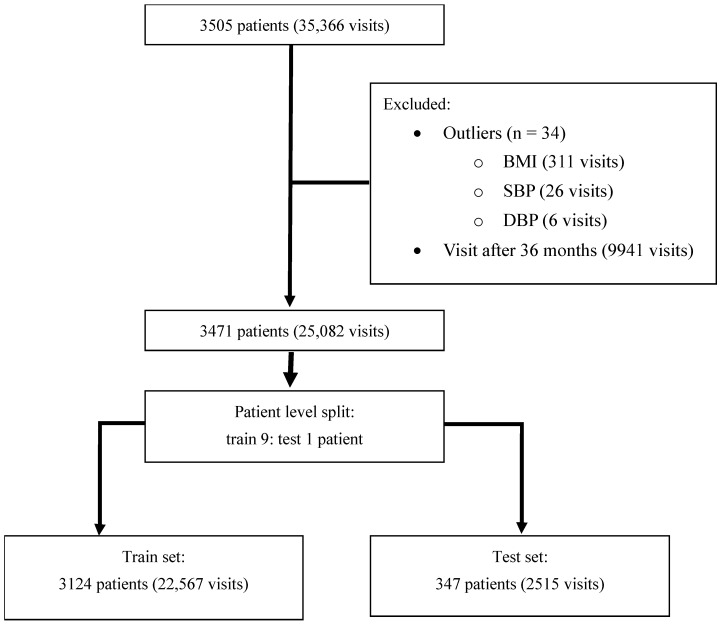
Flow diagram of the study population selection process. From an initial cohort of 3505 patients, 34 were excluded due to outlier data. Visits occurring after 36 months of follow-up were also removed, resulting in a final analytical cohort of 3471 patients (25,082 visits). This patient cohort was then divided at a 9:1 ratio into a training set and a testing set.

The study included data from patients aged 18–100 years. Initially, the records of 3505 patients were screened. We excluded 34 patients due to outlier data identified during exploratory data analysis, resulting in a final cohort of 3471 patients for the analysis. Within this cohort, follow-up visits exceeding 36 months were removed to focus the analysis on early-stage T2D. The final dataset was then divided into training and testing sets using a 9:1 ratio. This split was stratified by the patient’s final CKD status (eGFR < 60 vs. eGFR ≥ 60 mL/min/1.73 m^2^) to ensure a proportional representation of the outcome in both the training and testing sets. The patient selection process is summarized in Figure 1.

### 2.3. Feature Selection and Labeling

Features were chosen on the basis of expert advice and primary care hospital practicality, including age, gender, body mass index, T2D duration, systolic and diastolic blood pressures, fasting blood glucose (FBG) levels, and hemoglobin A1c (HbA1c) levels. Exact data on the T2D duration were lacking; therefore, for this study, we assumed that the first visit to the diabetic clinic was flagged when the diagnosis of T2D was established. In primary care, HbA1c testing was performed annually; therefore, we developed two models: one accounting for all features, including the HbA1c level (the “with-HbA1c” model), and the other excluding the HbA1c level (the “non–HbA1c” model) for broader applicability. The CKD status was assigned using an algorithmic approach based on the clinical definition of chronicity. A patient was labeled with CKD at the first visit when their eGFR was below 60 mL/min/1.73 m^2^, provided this was confirmed by an eGFR also meeting this criterion from a previous visit that occurred 90 days or more prior. Once a patient was diagnosed with CKD, this label was carried forward for all subsequent visits. The eGFR was calculated from serum creatinine using the Chronic Kidney Disease Epidemiology Collaboration equation.

### 2.4. Data and Null Value Management

To manage the dataset for this study, we carefully evaluated the null values for the selected features. Each patient visit was treated as a distinct data entry, and the following steps were taken for data cleaning and preparation: (1) We first assessed each feature for its completeness; features for which null values constituted >25% of the data were excluded from the analysis (Supplementary Materials, Table S1). This decision was made to ensure that our analysis relied on features with robust and reliable data. (2) For features for which null values constituted <25% of the data, we used a patient-specific approach to data imputation: in cases of missing data, we imputed the median value of that specific feature from the same patient. This method ensured that the individual characteristics and patterns of each patient were consistent, which thereby enhanced the accuracy and relevance of our datasets.

### 2.5. Model Development and Evaluation

In developing our models, we leveraged three principal tree-based ML algorithms:

1. The decision tree algorithm [20]: In this algorithm, the dataset is divided into subsets on the basis of different criteria, and at each internal node, a decision is made according to the value of a certain attribute. The “leaves” of the “tree” represent classifications or decisions. Because of its simplicity in design and interpretability, this model is useful for initial analysis.
2. The random forest algorithm [21]: In this ensemble method, multiple decision trees are constructed during the training phase. For classification tasks, the output is the mode of the classes (the class selected by the most trees), and for regression, it is the average prediction of the individual trees. The random forest method improves the model accuracy and overcomes the overfitting issue common with single-decision trees.
3. The extreme gradient boosting (XGBoost) algorithm [22]: This highly efficient and scalable implementation of gradient boosting improves upon the traditional boosting method by incorporating regularized learning to prevent overfitting, which makes the model robust in a variety of data scenarios. Its execution speed and model performance are attributable to its advanced handling of sparse data and ability to run on various hardware platforms.

For each of these algorithms, we fine-tuned the hyperparameters by using an exhaustive search for the decision tree algorithm and a randomized search (spanning 100 iterations) for the random forest and XGBoost algorithms. The performance of each algorithm in predicting the development of CKD was assessed through 10-fold cross-validation, with a focus on metrics such as the accuracy and macroaverage F1-score. The folds for this cross-validation were stratified by CKD status. To develop the non-HbA1c model, we used the best-performing algorithm from the “with-HbA1c” model.

We compared the models’ efficacies by using the area under the receiver operating characteristic curve (AUROC) from the test datasets, and we evaluated the performances across multiple metrics, including the precision, recall, accuracy, F1-score, sensitivity, specificity, positive predictive value (PPV), and negative predictive value (NPV) [23]. To quantify the statistical uncertainty of the performance metrics, 95% confidence intervals (CIs) were calculated for the accuracy, AUROC, macroaveraged F1-score, precision, and recall. These CIs were generated using the percentile bootstrap method, which involved resampling the test set with replacement 1000 times and calculating the 2.5th and 97.5th percentiles of the resulting distribution of scores for each metric. We assessed the model calibration by generating a calibration plot to compare the predicted probabilities against the observed frequencies of CKD on the test set. Predicted probabilities for the primary binary outcome generated by the best model were applied to the independent test set. Probabilities were stratified into four risk tiers at 0.25-probability increments. For each tier, we calculated the PPV and positive likelihood ratio (LR+). A Cochran–Armitage test for linear trends was applied to the four ordered risk groups to evaluate whether the event proportion increased with higher predicted-risk tiers.

To analyze the influence of individual features in the final model, we employed the SHAP (Shapley additive explanation) method. We utilized the TreeExplainer implementation from the SHAP library, which is specifically optimized for tree-based models. SHAP values were calculated for all patients in the training dataset to provide a comprehensive and global understanding of the model’s feature contributions [24].

### 2.6. Statistical Analysis

Continuous data were analyzed as means ± standard deviations or as medians with interquartile ranges (IQRs) according to distribution and were assessed through the use of histograms and the Shapiro–Wilk test. Categorical data were analyzed as frequencies. For inferential statistical comparisons between the first visit of patients with and without a CKD diagnosis, the Student’s *t*-test was used for normally distributed continuous variables, while the Mann–Whitney U test was used for non-normally distributed variables. The chi-squared (χ^2^) test or Fisher’s exact test, as appropriate, was used for categorical variables. A two-sided *p*-value of <0.05 was considered statistically significant for all tests. No corrections for multiple comparisons were applied to the baseline characteristic comparisons. For analysis and model development, we utilized Python version 3.10 on Google Colaboratory, with libraries that included NumPy, version 1.23.5; pandas, version 1.5.3; seaborn, version 0.12.2; scikit-learn, version 1.2.2; Matplotlib, version 3.7.1; SciPy, version 1.11.4; XGBoost, version 1.7.6; and SHAP, version 0.44.

## 3. Results

### 3.1. Dataset Characteristics

Our study initially included data from 3505 patients, which accounted for 35,366 visits. After the exclusion of visits beyond 36 months of individual follow-up and outliers, the analysis included data from 3471 patients, which accounted for 25,082 visits. We found no null values in the datasets. CKD was diagnosed in 10,833 (43.2%) of these visits. The training datasets involved 3124 patients (22,567 visits), the testing datasets involved 347 patients (2515 visits), and the proportions of CKD at the visit level were 43.6% in the training set and 39.9% in the test set. For the baseline characteristics at first visit, patients in the CKD group were significantly older (median age: 67.9 vs. 51.5 years, *p* < 0.0001) and had higher FBG (131 vs. 111 mg/dL, *p* < 0.0001) and HbA1c (7.1% vs. 6.3%, *p* < 0.0001) levels compared to the non-CKD group. A significantly lower proportion of female patients and lower diastolic blood pressure (DBP) were observed in the CKD group. Patients’ characteristics at first visit and at CKD diagnosis are summarized in Table 1. On average, the patients in our cohort had a mean of 2.5 HbA1c measurements per year.

### 3.2. Model Performance

**Figure 2:**
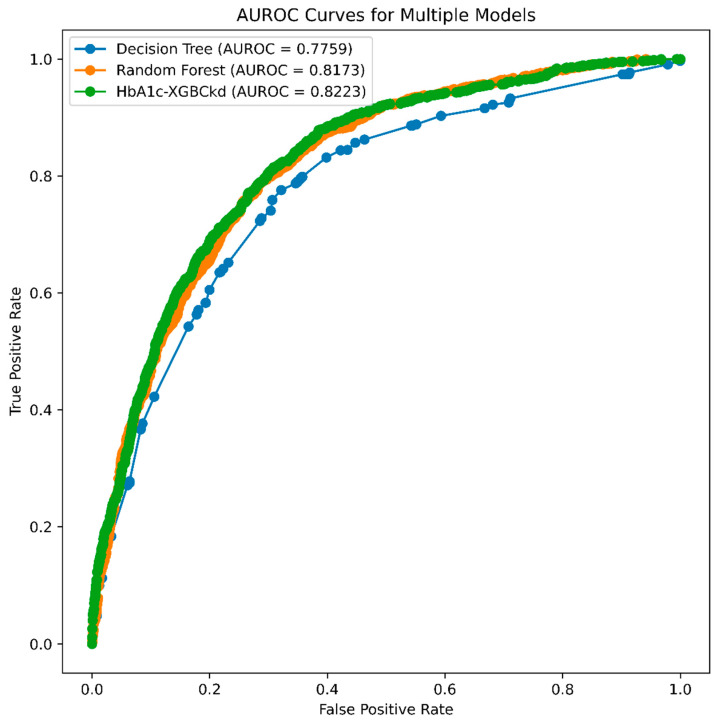
Area under the receiver operating characteristic (AUROC) curves for three different machine learning models: decision tree, random forest, and HbA1c-XGBCkd.

**Figure 3:**
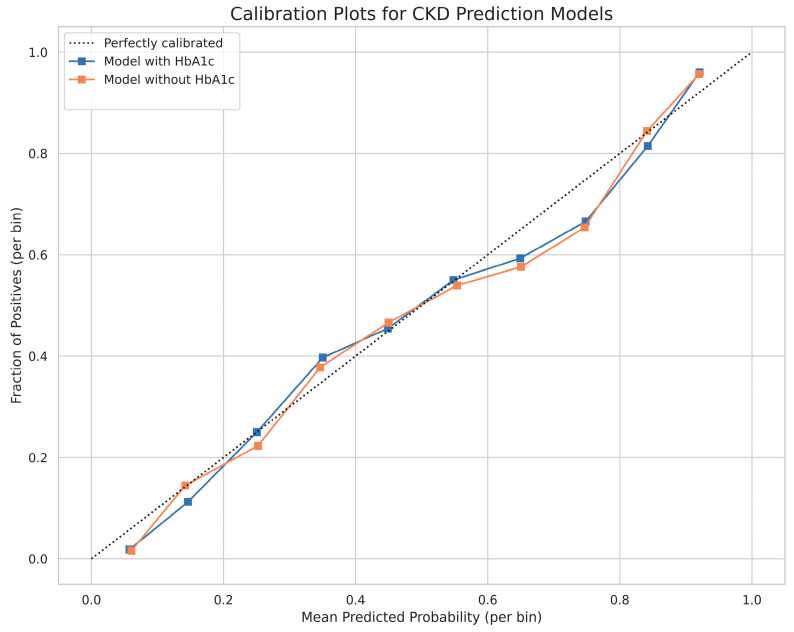
Calibration plots for the final models on the testing set. The plots compare the predicted probabilities of developing CKD against the actual observed frequencies. The diagonal dotted line represents perfect calibration.

The best-performing algorithm for the “with HbA1c” model was XGBoost (HbA1c-XGBckd), which achieved an AUROC of 0.824, slightly surpassing that of the random forest algorithm. The range and optimal hyperparameters are detailed in the Supplementary Materials, Table S2; the performance metrics are listed in Table 2. For the “non–HbA1c” model, the AUROC of the XGBoost algorithm (non–HbA1c-XGBckd), which was used to develop this model, was 0.819 (Figure 2). The calibration of the final models was assessed on the testing set (Figure 3). Both the model with HbA1c and the model without HbA1c demonstrated good calibration, with their predicted probabilities closely aligning with the observed frequencies of CKD across all risk strata.

### 3.3. Model Explanation

**Figure 4:**
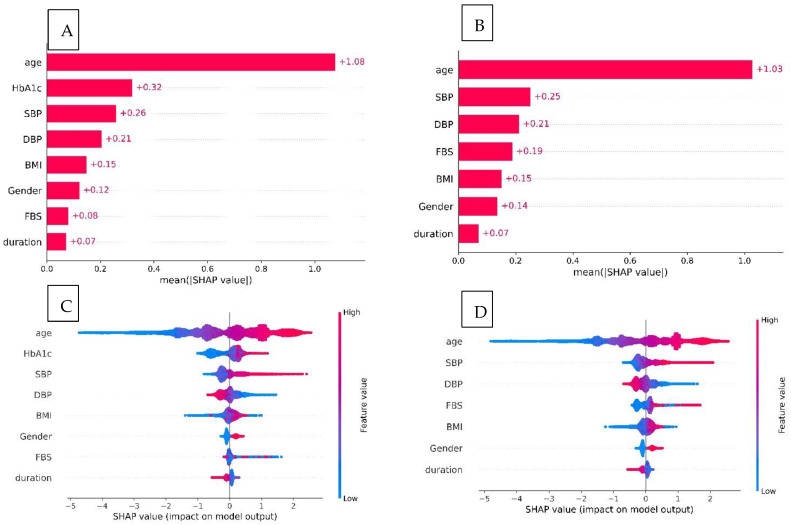
Importance matrix plots of the XGBoost model showing the importance of each variable for predicting chronic kidney disease: (**A**) HbA1c-XGBckd model; (**B**) non-HbA1c-XGBckd model. SHAP summary plots of the features: (**C**) HbA1c-XGBckd model; (**D**) non-HbA1c-XGBckd model.

**Figure 5:**
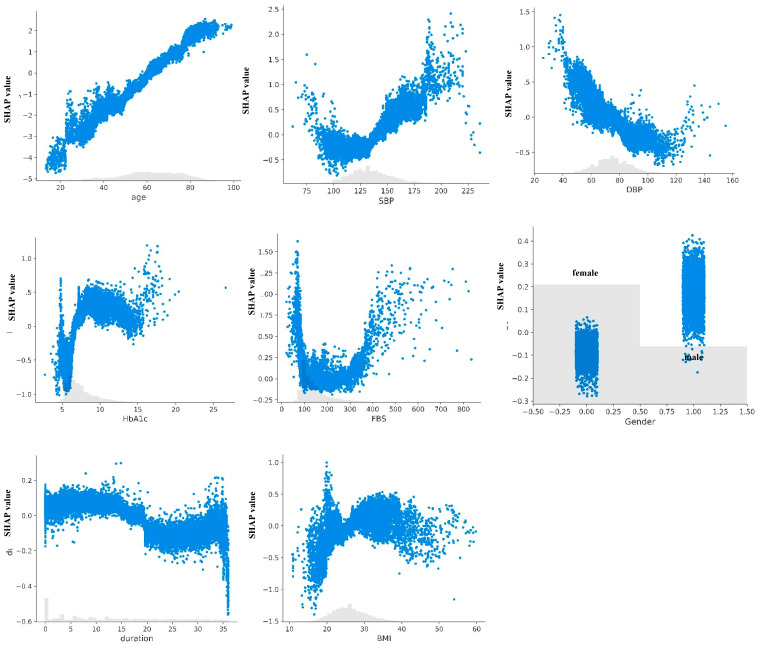
SHAP dependence plots of HbA1c-XGBckd model. Abbreviations: BMI, body mass index; DBP, diastolic blood pressure; FBS, fasting blood sugar; SBP, systolic blood pressure.

**Figure 6:**
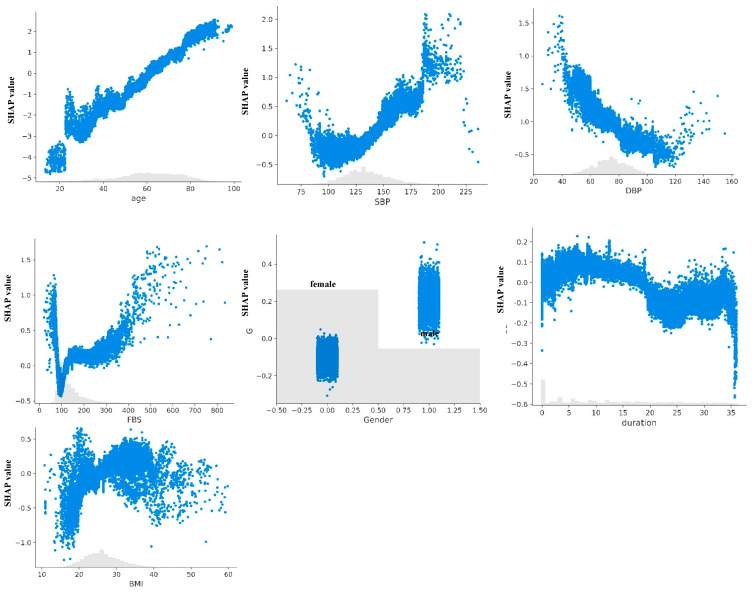
SHAP dependence plots of non-HbA1c-XGBckd model. Abbreviation: BMI, body mass index; DBP, diastolic blood pressure; FBS, fasting blood pressure; SBP, systolic blood pressure.

The SHAP value analysis for the HbA1c-XGBckd model (Figure 4) indicated that age, HbA1c levels, and systolic blood pressure (SBP) were the three features most predictive of the development of CKD. In the non–HbA1c-XGBckd model, the most predictive features were age, SBP, and DBP; also, the FBG level was associated with CKD diagnosis slightly more than in the HbA1c-XGBckd model. The SHAP dependence plots for each feature in both models, illustrated in Figure 5 and Figure 6, reveal a linear correlation of age with higher SHAP values. Notably, the plots for features such as the SBP, DBP, and FBG level exhibit a U-curve pattern. HbA1c values of >7% were associated with higher SHAP values, but they tended not to be any higher. Male gender was also correlated with increased SHAP values.

### 3.4. Risk Stratification Results

For the HbA1c-XGBckd model, the observed event rates increased steadily from 8.7% in the low-risk tier to 76.1% in the very-high-risk tier (Cochran–Armitage trend test, *p* < 0.001). The corresponding positive likelihood ratio rose from 0.16 to 5.46 (Table 3). The non-HbA1c-XGBckd model showed the same pattern: both the positive predictive value and likelihood ratio increased monotonically across the four risk strata, and the Cochran–Armitage test again indicated a highly significant linear trend (*p* < 0.001).

## 4. Discussion

In this study, we successfully developed and validated two simplified machine learning models for predicting the risk of reduced kidney function in a large retrospective cohort of Thai patients with T2D. Our primary finding is that the XGBoost algorithm, utilizing routinely collected clinical variables, demonstrated a strong discriminative performance. Crucially, the secondary model, developed without HbA1c, retained high accuracy and a strong AUROC, highlighting its potential as a highly accessible screening tool in diverse clinical settings where HbA1c data may not be readily available.

### 4.1. Clinical Interpretation of Model

A key strength of our study is the model’s interpretability, which we analyzed using the SHAP method. This analysis not only identified the most important predictors but also provided granular insights into how specific value ranges influence the CKD risk, aligning with established clinical knowledge [25,26].

The SHAP plots revealed that advanced age was the most powerful and consistent predictor, with the risk increasing linearly across the entire age spectrum. HbA1c was also a major driver, exhibiting a clear threshold effect where the risk of CKD began to rise sharply once the levels exceeded approximately 7%. This highlights the critical importance of maintaining glycemic control below this well-established target.

Interestingly, the model identified a U-shaped relationship for both SBP and FBG. For SBP, the risk was elevated at both low (<100 mmHg) and high (>140 mmHg) values, suggesting that both hypotension and hypertension contribute to kidney damage in this population. Similarly, both hypoglycemia (FBS < 100 mg/dL) and significant hyperglycemia (FBS > 200 mg/dL) increased the predicted risk. While the association with hyperglycemia is expected, the link between lower blood pressure, lower FBS, and higher risk could reflect underlying patient frailty or other comorbidities.

While a longer duration of T2D is a known risk factor for CKD [25], it did not emerge as a top predictor in our model, likely attributable to the relatively short follow-up period of our cohort. These detailed findings enhance the model’s trustworthiness by demonstrating that its internal logic is clinically plausible and rooted in established physiological principles.

### 4.2. Performance in Context and Clinical Utility

Our model’s performance, with an AUROC of 0.824, is comparable to or exceeds those of more complex models reported in the literature [27,28,29]. For example, Liu et al. achieved a similar AUROC of 0.827 using an XGBoost model on a Chinese dataset, but their model required more features and was developed on hospitalized patients, potentially limiting its generalizability to outpatient screening [30]. Other models, like that of Sammut-Powell et al., have shown more moderate performances with fewer variables [31,32]. However, it is important to contextualize these comparisons with the understanding that our study could not account for the use of renoprotective medications, which may have influenced the outcomes in our cohort and differed from the populations in other studies. In one notable study, Makino et al. used big data and deep learning techniques to predict the progression of diabetic kidney disease to stages 2–5 or stability at stage 1 within 6 months of diagnosis [33]. Their model, which included numerous numeric and text features, was trained with a complex deep learning algorithm and achieved an AUROC of 0.743. The concepts and outcomes of their study differed from those of our study; therefore, the performances of their model and ours are not comparable. Hence, our work fills a critical gap by achieving a strong performance with a minimal set of features readily available in primary care.

Beyond the AUROC, the clinical utility of a screening model hinges on the trade-off between sensitivity and specificity. For a screening tool intended for early detection, high sensitivity (recall) is paramount to minimize false negatives and avoid missing patients who could benefit from early intervention. Our model achieved an acceptable sensitivity, demonstrating its ability to correctly identify a large proportion of patients with true CKD. The consequence of a false positive in this context is a follow-up confirmatory test (e.g., UACR or eGFR), which is a low-cost and low-risk event compared to the consequence of a false negative—a missed opportunity to prevent or slow the progression to ESKD. Therefore, our model is well-suited for its intended purpose as a clinical screening tool.

### 4.3. Perspectives for Clinical Practice

The findings of this study offer two distinct but complementary pathways for implementation in clinical practice. The first pathway involves integrating the model into clinical decision support systems. We envision our model being embedded within a hospital’s Electronic Health Record (EHR) system. This system could automatically flag high-risk patients based on their routine data, alerting the physician during a consultation and prompting them to order confirmatory tests. Given that our model’s definition of CKD is based solely on the eGFR, its practical role would be to act as an effective trigger for more comprehensive testing, such as UACR analysis, rather than as a standalone diagnostic. This creates a practical, automated mechanism to improve adherence to KDIGO screening guidelines and facilitate timely referrals to specialist care. The second and more patient-centric pathway is the development of an mHealth application. A secure mobile app could allow patients with T2D to input their own data to receive an instant, personalized risk probability. This would empower patients by increasing their awareness of kidney health, encouraging proactive lifestyle modifications, and facilitating more informed discussions with their healthcare providers. Such a tool aligns with the growing trend of patient-centered care and could significantly enhance the self-management of diabetes and its complications. The simplicity of the non-HbA1c model makes both of these implementation strategies highly feasible, provided they are presented within the clear context of being an initial risk stratification tool.

### 4.4. Limitations and Strengths

This study has several limitations. A primary limitation relates to the generalizability of our findings. This was a single-center, retrospective study conducted at a university hospital in Thailand. The specific demographics, treatment patterns, and disease severity of our patient cohort may not be representative of the broader diabetic population in primary care settings or in other countries. Therefore, the model’s performance may differ in distinct clinical contexts. Second, our definition of CKD was based solely on the eGFR, as albuminuria data were not consistently available. This means that our model would not have captured patients with early-stage CKD who presented with normal eGFRs but met other diagnostic criteria, such as having significant albuminuria. Third, it should also be noted that our model relies on HbA1c as a key predictor, and its accuracy can be affected by certain conditions. We did not account for the potential influence of factors like anemia or advanced uremia, which are known to interfere with HbA1c measurements [34,35]. Fourth, we did not perform a formal sensitivity analysis to test the model’s robustness to input variation. Furthermore, our study is limited by the lack of medication data in the retrospective dataset. We were unable to account for the use of renoprotective therapies, particularly renin–angiotensin–aldosterone system (RAAS) blockers, which could have acted as a confounding factor by slowing the CKD progression in treated patients. While the impact of SGLT2 inhibitors is not a factor for this cohort, as they were not available in Thailand during the study period, the inability to adjust for RAAS blockade is a significant limitation that may affect the model’s generalizability to different populations with varying treatment patterns.

Despite these limitations, our study has significant strengths. Its main strength lies in the development of a simple, transparent, and strong-performing model using a small number of routine clinical variables. By validating our model in a specific Thai population, we provide a tool that is directly relevant to a region with a high burden of T2D.

## 5. Conclusions

In conclusion, our simplified XGBoost models demonstrate a strong performance and good calibration for predicting the risk of reduced kidney function in Thai patients with T2D. The development of these interpretable models represents a promising step toward integrating ML into clinical practice for CKD screening. However, it is crucial to emphasize that the model’s effectiveness needs to be confirmed in external and prospective contexts before it can be recommended for widespread clinical use. Furthermore, for this tool to be ethically and robustly implemented, future development must focus on enriching the model with crucial biomarkers, particularly albuminuria, and incorporating data on key therapeutic variables, such as the use of nephroprotective medications. This will ensure the creation of a more comprehensive and reliable tool for clinical decision making.

## Figures and Tables

**Table 1:** Patient characteristics at baseline visit according to CKD status during follow-up.

	Total	Non-CKD Group	CKD Group	*p*-Value
Number of patients	3471	1607	1864	
Age, year	59.9 (49.8, 70.4)	51.5 (41.9, 59.1)	67.9 (59.3, 75.1)	<0.0001
Gender, female	2367 (68.2%)	1125 (70.0%)	622 (66.6%)	0.0332
BMI, kg/m^2^	25.9 (22.7, 29.3)	25.8 (22.7, 29.8)	26.0 (22.9, 28.9)	0.3979
SBP, mmHg	132 (120, 145)	111 (95, 161)	131 (100, 171)	<0.0001
DBP, mmHg	75 (67, 83)	77 (69, 85)	74 (65, 81)	<0.0001
FBS, mg/dL	121 (98, 170)	111 (95, 161)	131 (100, 177)	<0.0001
HbA1c, %	6.7 (5.9, 8.3)	6.3 (5.7, 7.9)	7.1 (6.1, 8.5)	<0.0001

Data are presented as medians (quartile 1, quartile 3) for continuous variables or as n (%) for categorical variables. *p*-values were calculated using the Mann–Whitney U test for continuous variables and the chi-squared test for categorical variables.

**Table 2:** Results of model performance on training and testing dataset.

	Training Dataset				Testing Dataset				
	Accuracy	Precision	Recall	F1-Score	AUROC (95% CI)	Accuracy (95% CI)	Precision (95% CI)	Recall (95% CI)	F1-Score (95% CI)
Dataset with HbA1c data									
DT	0.73	0.73	0.73	0.73	0.781 (0.761–0.792)	0.71 (0.698–0.728)	0.70 (0.670–0.719	0.72 (0.704–0.735)	0.70 (0.689–0.729)
RF	0.81	0.81	0.81	0.81	0.818 (0.801–0.832)	0.75 (0.735–0.764)	0.73 (0.720–0.748)	0.74 (0.729–0.758)	0.74 (0.723–0.752)
XGB	0.77	0.77	0.77	0.77	0.824 (0.808–0.836)	0.75 (0.740–0.768)	0.74 (0.724–0.751)	0.75 (0.731–0.761)	0.74 (0.726–0.755)
Dataset without HbA1c data									
XGB	0.76	0.76	0.76	0.76	0.819 (0.804–0.833)	0.75 (0.737–0.765)	0.74 (0.723–0.751)	0.75 (0.735–0.764)	0.74 (0.727–0.755)

Abbreviations: AUROC, area under the receiver operating characteristic curve; DT, decision tree model; HbA1c, glycated hemoglobin A1c; RF, random forest model; XGB, extreme gradient boosting model; 95% CI, 95% confidence interval.

**Table 3:** Risk stratification results.

Model	Risk Tier	Subjects	Events	PPV (95% CI)	LR+ (95% CI)	Cochran–Armitage Test
HbA1c-XGBckd						<0.001
	Low	1268	110	0.087 (0.071–0.102)	0.16 (0.13–0.20)	
	Moderate	839	261	0.311 (0.280–0.342)	0.77 (0.66–0.91)	
	High	836	477	0.571 (0.537–0.604)	2.28 (1.95–2.65)	
	Very high	603	459	0.761 (0.727–0.795)	5.46 (4.47–6.66)	
Non-HbA1c-XGBckd						<0.001
	Low	1252	103	0.083 (0.067–0.097)	0.15 (0.12–0.19)	
	Moderate	775	232	0.299 (0.267–0.332)	0.73 (0.62–0.87)	
	High	916	523	0.571 (0.539–0.603)	2.28 (1.97–2.64)	
	Very high	603	449	0.745 (0.710–0.779)	4.99 (4.11–6.07)	

Abbreviation: LR+, positive likelihood ratio; PPV, positive predictive value.

## Data Availability

The datasets used and/or analyzed during the current study are available from the corresponding author upon reasonable request.

## Supplementary Materials

The following supporting information can be downloaded at: https://www.mdpi.com/article/10.3390/jcm14134735/s1, Table S1: Feature selection based on data completeness; Table S2: Hyperparameter optimization space and final selected values for each model; Table S3: Software libraries used for data analysis and model development.

## Abbreviations

AUROC	Area under the receiver operating characteristic curve
CKD	Chronic kidney disease
CVD	Cardiovascular disease
DBP	Diastolic blood pressure
eGFR	Estimated glomerular filtration rate
ESKD	End-stage kidney disease
FBG	Fasting blood glucose
HbA1c	Glycated hemoglobin
ICD	International Classification of Diseases
LR+	Positive likelihood ratio
mHealth	Mobile health
ML	Machine learning
NPV	Negative predictive value
PPV	Positive predictive value
RAAS	Renin–angiotensin–aldosterone system
SBP	Systolic blood pressure
SHAP	Shapley additive explanations
T2D	Type 2 diabetes
XGBoost	Extreme gradient boosting

## References

[1] Shah A., Isath A., Aronow W.S. (2022). Cardiovascular complications of diabetes. Expert. Rev. Endocrinol. Metab..

[2] Zakir M., Ahuja N., Surksha M.A., Sachdev R., Kalariya Y., Nasir M., Kashif M., Shahzeen F., Tayyab A., Khan M.S.M. (2023). Cardiovascular Complications of Diabetes: From Microvascular to Macrovascular Pathways. Cureus.

[3] Hauwanga W.N., Abdalhamed T.Y., Ezike L.A., Chukwulebe I.S., Oo A.K., Wilfred A., Khan A., Chukwuwike J., Florial E., Lawan H. (2024). The Pathophysiology and Vascular Complications of Diabetes in Chronic Kidney Disease: A Comprehensive Review. Cureus.

[4] Rossing P., Caramori M.L., Chan J.C., Heerspink H.J., Hurst C., Khunti K., Liew A., Michos E.D., Navaneethan S.D., Olowu W.A. (2022). KDIGO 2022 Clinical Practice Guideline for Diabetes Management in Chronic Kidney Disease. Kidney Int..

[5] Stevens P.E., Ahmed S.B., Carrero J.J., Foster B., Francis A., Hall R.K., Herrington W.G., Hill G., Inker L.A., Kazancıoğlu R. (2024). KDIGO 2024 Clinical Practice Guideline for the Evaluation and Management of Chronic Kidney Disease. Kidney Int..

[6] Bikbov B., Purcell C.A., Levey A.S., Smith M., Abdoli A., Abebe M., Adebayo O.M., Afarideh M., Agarwal S.K., Agudelo-Botero M. (2020). Global, regional, and national burden of chronic kidney disease, 1990–2017: A systematic analysis for the Global Burden of Disease Study 2017. Lancet.

[7] Jankowski J., Floege J., Fliser D., Böhm M., Marx N. (2021). Cardiovascular Disease in Chronic Kidney Disease: Pathophysiological Insights and Therapeutic Options. Circulation.

[8] Aekplakorn W., Chariyalertsak S., Kessomboon P., Assanangkornchai S., Taneepanichskul S., Putwatana P. (2018). Prevalence of Diabetes and Relationship with Socioeconomic Status in the Thai Population: National Health Examination Survey, 2004–2014. J. Diabetes Res..

[9] Nata N., Rangsin R., Supasyndh O., Satirapoj B. (2020). Impaired Glomerular Filtration Rate in Type 2 Diabetes Mellitus Subjects: A Nationwide Cross-Sectional Study in Thailand. J. Diabetes Res..

[10] Jha V., Al-Ghamdi S.M.G., Li G., Wu M.S., Stafylas P., Retat L., Card-Gowers J., Barone S., Cabrera C., Garcia Sanchez J.J. (2023). Global Economic Burden Associated with Chronic Kidney Disease: A Pragmatic Review of Medical Costs for the Inside CKD Research Programme. Adv. Ther..

[11] Park J.I., Baek H., Jung H.H. (2016). CKD and Health-Related Quality of Life: The Korea National Health and Nutrition Examination Survey. Am. J. Kidney Dis..

[12] Sguanci M., Mancin S., Gazzelloni A., Diamanti O., Ferrara G., Morales Palomares S., Parozzi M., Petrelli F., Cangelosi G. (2024). The Internet of Things in the Nutritional Management of Patients with Chronic Neurological Cognitive Impairment: A Scoping Review. Healthcare.

[13] Kitsiou S., Gerber B.S., Buchholz S.W., Kansal M.M., Sun J., Pressler S.J. (2025). Patient-Centered mHealth Intervention to Improve Self-Care in Patients With Chronic Heart Failure: Phase 1 Randomized Controlled Trial. J. Med. Internet Res..

[14] Gerber B.S., Biggers A., Tilton J.J., Smith Marsh D.E., Lane R., Mihailescu D., Lee J., Sharp L.K. (2023). Mobile Health Intervention in Patients With Type 2 Diabetes: A Randomized Clinical Trial. JAMA Netw. Open.

[15] Kitsiou S., Paré G., Jaana M., Gerber B. (2017). Effectiveness of mHealth interventions for patients with diabetes: An overview of systematic reviews. PLoS ONE.

[16] Waki K., Nara M., Enomoto S., Mieno M., Kanda E., Sankoda A., Kawai Y., Miyake K., Wakui H., Tsurutani Y. (2024). Effectiveness of DialBetesPlus, a self-management support system for diabetic kidney disease: Randomized controlled trial. npj Digit. Med..

[17] Haug C.J., Drazen J.M. (2023). Artificial Intelligence and Machine Learning in Clinical Medicine, 2023. N. Engl. J. Med..

[18] Mesquita F., Bernardino J., Henriques J., Raposo J.F., Ribeiro R.T., Paredes S. (2024). Machine learning techniques to predict the risk of developing diabetic nephropathy: A literature review. J. Diabetes Metab. Disord..

[19] Sabanayagam C., He F., Nusinovici S., Li J., Lim C., Tan G., Cheng C.Y. (2023). Prediction of diabetic kidney disease risk using machine learning models: A population-based cohort study of Asian adults. elife.

[20] Quinlan J.R. (1986). Induction of decision trees. Mach. Learn..

[21] Breiman L. (2001). Random Forests. Mach. Learn..

[22] Chen T., Guestrin C. XGBoost: A Scalable Tree Boosting System. Proceedings of the 22nd ACM SIGKDD International Conference on Knowledge Discovery and Data Mining.

[23] Powers D. (2008). Evaluation: From Precision, Recall and F-Factor to ROC, Informedness, Markedness & Correlation. Mach. Learn. Technol..

[24] Lundberg S.M., Lee S.-I. A Unified Approach to Interpreting Model Predictions. Proceedings of the 31st International Conference on Neural Information Processing Systems.

[25] Siddiqui K., George T.P., Joy S.S., Alfadda A.A. (2022). Risk factors of chronic kidney disease among type 2 diabetic patients with longer duration of diabetes. Front. Endocrinol..

[26] Harjutsalo V., Groop P.H. (2014). Epidemiology and risk factors for diabetic kidney disease. Adv. Chronic Kidney Dis..

[27] Abdel-Fattah M.A., Othman N.A., Goher N. (2022). Predicting Chronic Kidney Disease Using Hybrid Machine Learning Based on Apache Spark. Comput. Intell. Neurosci..

[28] Iparraguirre-Villanueva O., Espinola-Linares K., Flores Castañeda R.O., Cabanillas-Carbonell M. (2023). Application of Machine Learning Models for Early Detection and Accurate Classification of Type 2 Diabetes. Diagnostics.

[29] Dong Z., Wang Q., Ke Y., Zhang W., Hong Q., Liu C., Liu X., Yang J., Xi Y., Shi J. (2022). Prediction of 3-year risk of diabetic kidney disease using machine learning based on electronic medical records. J. Transl. Med..

[30] Liu X.Z., Duan M., Huang H.D., Zhang Y., Xiang T.Y., Niu W.C., Zhou B., Wang H.L., Zhang T.T. (2023). Predicting diabetic kidney disease for type 2 diabetes mellitus by machine learning in the real world: A multicenter retrospective study. Front. Endocrinol..

[31] Sammut-Powell C., Sisk R., Silva-Tinoco R., de la Pena G., Almeda-Valdes P., Juarez Comboni S.C., Goncalves S., Cameron R. (2024). External validation of a minimal-resource model to predict reduced estimated glomerular filtration rate in people with type 2 diabetes without diagnosis of chronic kidney disease in Mexico: A comparison between country-level and regional performance. Front. Endocrinol..

[32] Sammut-Powell C., Sisk R., Vazquez-Mendez E., Vasnawala H., Goncalves S., Edge M., Cameron R. (2024). Global Validation of a Model to Predict Reduced Estimated GFR in People With Type 2 Diabetes Without Diagnosis of CKD. Kidney Int. Rep..

[33] Makino M., Yoshimoto R., Ono M., Itoko T., Katsuki T., Koseki A., Kudo M., Haida K., Kuroda J., Yanagiya R. (2019). Artificial intelligence predicts the progression of diabetic kidney disease using big data machine learning. Sci. Rep..

[34] Katwal P.C., Jirjees S., Htun Z.M., Aldawudi I., Khan S. (2020). The Effect of Anemia and the Goal of Optimal HbA1c Control in Diabetes and Non-Diabetes. Cureus.

[35] Cavagnolli G., Pimentel A.L., Freitas P.A., Gross J.L., Camargo J.L. (2015). Factors affecting A1C in non-diabetic individuals: Review and meta-analysis. Clin. Chim. Acta.

