# Supplementary material for: A Simplified Machine Learning Model for Predicting Reduced Kidney Function in Thai Patients with Type 2 Diabetes: A Retrospective Study
Source: J Clin Med. 2025 Jul 4;14(13):4735. doi: 10.3390/jcm14134735 (PMC12250820; doi:10.3390/jcm14134735)
Supplement: Supplementary file 1 [file jcm-14-04735-s001.zip › jcm-3670668-supplementary.pdf]

Supplementary Materials

Supplementary Table S1. Feature selection based on data completeness.

Included features		Excluded features	
Features	Missing (%)	Features	Missing (%)
Gender	0	Urine albumin-creatinine ratio	87.2
Age	0	Low-density lipoprotein	74.3
Duration of diabetes	0	High-density lipoprotein	26.0
Body weight	0	Pulse rate	29.5
Height	0		
Systolic blood pressure	0		
Diastolic blood pressure	0		
FBS	0		
HbA1c	0		
Creatinine	0		
Cholesterol	0		
Triglyceride	0		

This table lists the clinical variables considered for model inclusion. Features with more than 25% missing values across the entire dataset were excluded from the analysis to ensure model robustness and avoid potential imputation bias. All included features had 0% missing values.

Supplementary Table S2. Hyperparameter optimization space and final selected values for each model.

Model	Hyperprameters	Range	Final model
DT	Criterion<br>Max depth<br>Min samples split<br>Min samples leaf<br>Max features<br>Min impurity decrease<br>Ccp alpha	Gini, Entropy<br>4,6,8,10<br>2, 4<br>1, 2, 4<br>Sqrt, Log2<br>0.0, 0.1<br>0.0, 0.1	Entropy<br>6<br>2<br>1<br>Log2<br>0.0<br>0.0
RF	N estimators<br>Max depth<br>Min samples split<br>Min samples leaf<br>Max features<br>Min impurity decrease<br>Ccp alpha	100, 200, 400<br>6, 10, 14<br>2, 4<br>1, 2, 4<br>Sqrt, Log2<br>0.0, 0.1<br>0.0, 0.1	200<br>10<br>2<br>2<br>Sqrt<br>0.0<br>9.9
HbA1c-XGBckd	N estimators<br>Learning rate<br>Colsample_bytree<br>Subsample<br>Max depth<br>Reg_lambda<br>Gamma	100, 300, 500<br>0.1, 0.01<br>0.6, 1.0<br>0.6, 0.8, 1.0<br>4, 8, 12<br>0, 0.1, 0.01<br>0, 0.1, 0.01	100<br>0.1<br>0.6<br>0.6<br>4<br>0.01<br>0.1
Non-HbA1c-XGBckd	N estimators<br>Learning rate<br>Colsample_bytree<br>Subsample<br>Max depth	100, 300, 500<br>0.1, 0.01<br>0.6, 1.0<br>0.6, 0.8, 1.0<br>4, 8, 12	100<br>0.1<br>0.6<br>0.8<br>4

	Reg_lambda<br>Gamma	0, 0.1, 0.01<br>0, 0.1, 0.01	0<br>0.01

The table details the search space used for hyperparameter tuning and the final optimal parameters selected for each algorithm. An exhaustive search was used for the Decision Tree, while a randomized search (100 iterations) was used for the Random Forest and XGBoost models.

Abbreviations: DT, Decision Tree; RF, Random Forest; HbA1c-XGBckd, XGBoost model with HbA1c; Non-HbA1c-XGBckd, XGBoost model without HbA1c; Ccp_alpha, Complexity Parameter Alpha.

Supplementary Table S3. Software libraries used for data analysis and model development.

Libraries	Usage
NumPy	Utilized for fundamental numerical operations, including the efficient handling of arrays and mathematical functions required for data preprocessing and analysis.
Pandas	Employed for all data manipulation tasks. Its DataFrame structure was used to load, clean, wrangle, and structure the dataset prior to analysis and model training.
Scikit-learn	• Splitting the dataset into stratified training and testing sets (train_test_split).<br>• Implementing the Decision Tree and Random Forest algorithms.<br>• Tuning model hyperparameters (RandomizedSearchCV, GridSearchCV).<br>• Calculating performance evaluation metrics (e.g., AUROC, F1-score, precision, recall, classification_report).<br>• Generating the calibration curve data (calibration_curve)
SciPy	Used for performing the formal inferential statistical tests reported in the study, including the Shapiro-Wilk test for normality, Student's t-test, Mann-Whitney U test, and the chi-squared test.
XGBoost	Provided the implementation of the Extreme Gradient Boosting algorithm.
Matplotlib and Seaborn	Used in combination for all data visualizations. Matplotlib provided the foundational plotting framework, while seaborn was used to create aesthetically pleasing and informative statistical graphics, such as histograms and the final calibration plots.
SHAP	Employed for model interpretation. This library was used to calculate Shapley values to explain the output of the final XGBoost model and to visualize the impact of each feature on the prediction of CKD risk.

The analysis was conducted using Python (version 3.10) in the Google Colaboratory environment. This table describes the primary libraries and their specific roles in the study.

Abbreviations: AUROC, Area Under the Receiver Operating Characteristic Curve; SHAP, Shapley Additive Explanations.
